# The association between worry, rumination, and metacognitive beliefs in female adolescents with Anorexia Nervosa

**DOI:** 10.3389/fpsyt.2026.1812206

**Published:** 2026-07-22

**Authors:** Anna Guerrini Usubini, Adele Bondesan, Diana Caroli, Francesca Frigerio, Laura Abbruzzese, Caterina Deodato, Gianluca Castelnuovo, Alessandro Sartorio

**Affiliations:** 1 Experimental Laboratory for Auxo-Endocrinological Research, Istituto Auxologico Italiano, Istituto di Ricovero e Cura a Carattere Scientifico (IRCCS), Piancavallo-Verbania, Italy; 2 Division of Auxology, Istituto Auxologico Italiano, Istituto di Ricovero e Cura a Carattere Scientifico (IRCCS), Piancavallo-Verbania, Italy; 3 Psychology Research Laboratory, Istituto Auxologico Italiano, Istituto di Ricovero e Cura a Carattere Scientifico (IRCCS), Piancavallo-Verbania, Italy; 4 Department of Psychology, Catholic University, Milan, Italy

**Keywords:** adolescents, anorexia nervosa, eating disorders, metacognitions, metacognitive beliefs, repetitive negative thinking, rumination, worry

## Abstract

**Background:**

A growing body of literature has emphasized the role of worry and rumination in eating disorders (EDs). According to the metacognitive model, worry and rumination are maintained by dysfunctional metacognitive beliefs. Although metacognitive beliefs have been investigated in adults with EDs, their role in adolescents with EDs remains underexplored. The present exploratory study aimed to examine the specific association among worry, rumination, and metacognitive beliefs and eating disorder symptomatology in adolescents with AN.

**Methods:**

Thirty Italian female adolescents (mean age ± SD: 15.387 ± 1.35 years, mean Body Mass Index (BMI): 16.34 ± 2.5 kg/m^2^) completed a self-report survey composed of the Penn State Worry Questionnaire (PSWQ), the Ruminative Response Scale (RRS), the Metacognitions Questionnaire – 30 (MCQ-30), and the Eating Disorder Inventory – 3 (EDI-3).

**Results:**

Thus, Model 1, including RRS and PSWQ, was significant (*F* = 17.5, *p<*0.001) and explained 57% of the variance (*R²* = 0.574). Within this model, RRS was the only significant predictor (*p* = 0.019) of the Eating Disorder Risk Composite (EDRC) of EDI-3, indicating that higher rumination scores were associated with greater eating disorder symptomatology. Once MCQ was entered as a predictor of EDRC, there was no significant incremental variance compared with Model 2 (Δ*R²* = 0.0034, *p* = 0.658), even though the model remained significant overall (*F* = 11.4, *p* < 0.001). The final model explained 58% of variance (*R²* = 0.578; Cohen’s *f^2^* = 1.37).

**Conclusion:**

Rumination was significantly associated with eating disorder symptomatology. Although worry showed strong positive associations with eating disorder symptomatology at the correlational level, it did not maintain an independent role once rumination was included in the regression model. The final model showed a very large effect size (Cohen’s f² = 1.37), suggesting strong associations between predictors and eating disorder symptoms in the present sample. However, this estimate should be interpreted cautiously, given the limited sample size and the exploratory nature of the analysis. Contrary to our expectations, metacognitive beliefs did not significantly explain the variance of eating disorder symptomatology. This finding may reflect the more proximal role of RNT in explaining the variance in eating disorder symptomatology, or alternatively, the possibility that only specific metacognitive domains—rather than overall metacognitive functioning—are relevant in this population.

## 1 Introduction

Eating disorders (EDs) are among the most severe psychiatric conditions, due to their high morbidity and mortality and their detrimental impact on both physical and mental health (1). Although EDs encompass a range of diagnostic presentations, Anorexia Nervosa (AN) is widely considered the most severe and clinically complex subtype. It is associated with marked psychological distress, serious medical complications, and one of the highest mortality rates of all psychiatric illnesses (2). Epidemiological evidence highlighted that AN typically emerge during adolescence and that its prevalence and clinical burden have increased in recent years. A recent comprehensive review (3) estimated the lifetime prevalence of AN to range from 0.8% to 6.3%. Specifically, prevalence estimates ranged from 0% to 3.2% in young women, while current prevalence rates in young men ranged from 0% to 1.6%.

A growing body of literature has emphasized the role of maladaptive cognitive processes in EDs (4, 5). Repetitive negative thinking (RNT) is a transdiagnostic construct encompassing persistent, recurrent, and difficult-to-control patterns of thinking, such as worry and rumination (6–8). Worry is defined as an uncontrollable chain of thoughts and images accompanied by negative emotions (9). It reflects an effort to solve problems whose outcomes are uncertain but could be mentally adverse. Rumination refers to repetitive patterns of thinking that focus on negative emotions and symptoms, as well as their causes, meanings, and consequences (10). While worry is generally oriented toward problem-solving and future possibilities, rumination more often is focused on past experiences (11).

Worry and rumination have been conceptualized as transdiagnostic maintenance mechanisms that contribute to the persistence and severity of EDs psychopathology (4). Specifically, it has been suggested that worry and rumination may interfere with adaptive emotional processing by promoting prolonged cognitive engagement with distressing thoughts while limiting emotional awareness and regulation (12). Within this framework, in AN, higher levels of worry and rumination may intensify preoccupations with body weight, shape, and control, thereby reinforcing psychological distress and maladaptive eating behaviors.

According to the metacognitive model (13, 14), worry and rumination are maintained by dysfunctional metacognitive beliefs that concern the usefulness, uncontrollability, and danger of one’s own thoughts (15). Previous research has shown a strong association between metacognitive beliefs and EDs (16, 17). It has been found that individuals with EDs tend to show high levels of positive metacognitive beliefs about worry as a useful coping strategy, along with low confidence in their cognitive abilities, such as memory and attention (18). In addition, they also reported elevated negative metacognitive beliefs concerning the uncontrollability and danger of worrying (18, 19). In this context, preoccupations with body weight and shape and fears of weight gain can be conceptualized as typical worry-related thoughts perceived as uncontrollable and harmful (16–18). Individuals with EDs also reported significantly higher levels of need to control thoughts (20–22). These beliefs indicate a tendency to excessively monitor and regulate one’s cognitive processes to prevent negative outcomes and have been conceptualized as reflecting a perceived lack of control over external events or internal states (16).

Despite this growing literature, important gaps remain. Research on metacognitive beliefs in EDs has predominantly focused on adult samples (16–23), while their role during adolescence—a key developmental window for the emergence and consolidation of ED psychopathology—remains largely underexplored. For instance, Limbers et al. (24) found that adolescents with high emotional eating reported higher levels of negative metacognitive beliefs compared to those with low emotional eating. Similarly, Guerrini Usubini et al. (25) showed that adolescents with obesity and comorbid binge eating disorder reported greater worry, rumination, and negative metacognitive beliefs than adolescents with obesity alone. While informative, these findings do not clarify whether and how metacognitive processes operate in adolescents with a full-threshold diagnosis of AN, nor do they address disorder-specific patterns of metacognition.

Given the early onset, severity, and developmental sensitivity of AN, investigating metacognitive beliefs and worry and rumination processes in adolescents with AN is crucial. Adolescence represents a particularly vulnerable phase in which maladaptive metacognitive styles may consolidate and contribute to the persistence of restrictive eating behaviors of AN.

Thus, the present exploratory study aimed to examine the specific association among worry, rumination, and metacognitive beliefs and eating disorder symptomatology in adolescents with AN. Specifically, we were interested in investigating the potential relationship between types of RNT (worry and rumination), metacognitive processes and eating disorder symptoms, in line with metacognitive models (13, 14) suggesting that maladaptive beliefs about cognition associated with perseverative thinking patterns may contribute to the maintenance of eating disorders.

For these reasons, in our exploratory study, we hypothesized that worry, rumination, and metacognitive beliefs would be significantly related to eating disorder symptomatology. In addition, worry, rumination, and metacognitive beliefs are expected to significantly account for a portion of variance in eating disorder symptomatology.

## 2 Material and methods

### 2.1 Participants and procedures

Participants in this cross-sectional, exploratory study were 30 consecutive Italian female adolescents diagnosed with AN (mean age ± SD: 15.9 ± 1.3 years; mean body mass index: BMI ± SD: 16.3 ± 2.5 kg/m²). Participants were recruited at the Division of Auxology, Istituto Auxologico Italiano, IRCCS, Piancavallo-Verbania, a third-level medical center for severe obesity and eating disorders, where the patients were hospitalized for a multidisciplinary rehabilitation. Inclusion criteria were (1): female gender (2); age between 11 and 17 years; and (3) a clinical diagnosis of AN according to the medical records. Diagnosis was established through a clinical interview, based on the Diagnostic and Statistical Manual of Mental Disorders, 5th Edition (DSM-5 (26);. The interview was conducted by a licensed clinical psychologist with expertise in the diagnosis and treatment of obesity and eating disorders, in accordance with the medical center’s internal procedure. Exclusion criteria included severe physical limitations (such as visual blindness) or mental impairments (including comorbid psychiatric diagnoses, as documented in medical records) that could interfere with study participation. After providing detailed information about the study, written informed consent was obtained from parents, and adolescents provided assent. An independent clinical psychologist with expertise in adolescent psychology conducted the screening interview to confirm eligibility. All participants met the inclusion criteria and were subsequently enrolled.

Once enrolled, participants provided socio-demographic information and completed self-report questionnaires.

The study was approved by the Territorial Ethical Committee (EC) no. 5, Lombardy region, Italy (EC code: 199/24, date of approval: April 23, 2024; order code: 01C412; acronym: METAED) and was conducted in accordance with the Declaration of Helsinki and its subsequent amendments.

### 2.2 Measures

Socio-demographic data: participants were asked to provide socio-demographic information (e.g., age, education, and region of origin).

Anthropometric data: the internal medical staff measured weight and height to calculate Body Mass Index (BMI) using the proper formula (kg/m^2^). Standing height was determined by a Harpenden Stadiometer (Holtain Limited, Crymych, Dyfed, UK). Weight was measured to the nearest 0.1 kg using an electronic scale (RoWU 150, Wunder Sa.bi., Trezzo sull’Adda, Italy).

Worry: the Penn State Worry Questionnaire (PSWQ (27, 28); is a self-report questionnaire designed to assess the general tendency to engage in excessive and uncontrollable worry. It consists of 16 items rated on a 5-point Likert scale (1 = not at all typical of me, 5 = very typical of me), with higher scores indicating greater levels of pathological worry. The PSWQ has demonstrated excellent internal consistency, test-retest reliability, and construct validity across clinical and non-clinical populations. In the present study, Cronbach’s alpha was 0.896.

Rumination: the Ruminative Responses Scale (RRS; 10 (29); is a self-report questionnaire designed to assess the tendency to repeatedly focus on one’s depressive symptoms, their causes, and their consequences. It captures a maladaptive pattern of self-focused rumination that has been linked to the onset and maintenance of depressive and emotional symptoms. The scale consists of 22 items rated on a Likert scale (typically 1 = almost never to 4 = almost always), with higher scores indicating greater levels of rumination. In the present study, Cronbach’s alpha was 0.931.

Metacognitions: the Metacognitions Questionnaire-30 (MCQ-30 (30, 31): is a self-report questionnaire designed to assess metacognitive beliefs and processes. It consists of 30 items rated on a 4-point Likert scale. The MCQ-30 assesses five dimensions of metacognition (1): positive beliefs about worry (e.g., beliefs that worrying is useful) (2), negative beliefs concerning the uncontrollability and danger of thoughts (3), cognitive confidence (i.e., confidence in memory and attentional abilities) (4), beliefs about the need to control thoughts, and (5) cognitive self-consciousness, reflecting the tendency to closely monitor one’s own thinking processes. Higher scores indicate more maladaptive metacognitive beliefs. The MCQ-30 has demonstrated good internal consistency, test-retest reliability, and construct validity in both clinical and non-clinical populations. In the present study, Cronbach’s alpha was 0.823.

Eating disorder symptomatology: the Eating Disorder Risk (EDRC) is one of the composite scales of the EDI-3 (Eating Disorder Inventory – 3rd edition (32);, that is used as an overall measure of the severity of eating disorder symptoms. It is calculated by combining three primary subscales of the EDI 3: Drive for Thinness (DT), which assesses excessive concern with body weight and fear of gaining weight; Bulimia (B), which measures tendencies toward binge eating and compensatory behaviors; Body Dissatisfaction (BD), which assesses dissatisfaction with specific body areas (e.g., abdomen, hips, thighs). The EDRC provides an overall estimate of eating disorder symptomsby capturing weight concerns, disordered eating behaviors, and body dissatisfaction. In the present study, Cronbach’s alpha of EDRC was 0.933.

### 2.3 Statistical analysis

An *a priori* power analysis for a linear regression model (α = 0.05, effect size f² = 0.35, and three predictors) indicated that a minimum sample of approximately N = 36 participants would be required to achieve 80% power.

Means and standard deviations, or frequencies and percentages, were computed to provide descriptive statistics. Skewness and Kurtosis were calculated to assess the normal distribution of the variables, and Cronbach’α was examined to assess the internal consistency of the scales. Preliminary associations between the study variables were performed using Pearson’s r correlation coefficient. Then, a hierarchical regression model was tested to assess whether worry, rumination, and metacognitive beliefs significantly accounted for variance in eating disorder symptomatology (EDRC). In the model, the EDRC served as the dependent variable. Predictors were entered in blocks: PSWQ and RRS in Step 1 and MCQ in Step 2. PSWQ and RRS were entered in Step 1 because they assess well-established cognitive processes (worry and rumination) that have consistently been associated with the outcome variable. MCQ was entered in Step 2 to examine whether metacognitive beliefs explain additional variance beyond these established cognitive factors. Cohen’s f2 effect size has been calculated. Data were analyzed using Jamovi (2.6.26).

## 3 Results

### 3.1 Descriptive statistics of the sample

We originally recruited 38 participants; however, individuals who did not complete the questionnaires were excluded from the analyses (n = 6). Additionally, two participants withdrew from the study after providing informed consent and completing the questionnaires. The final sample, therefore, consisted of 30 Italian female adolescent participants. In total, 83% lived in the North of Italy, 80% lived with both of their parents. Most of the participants (86%) were undergoing pharmacological treatments, including SSRI and antipsychotic medications. Descriptive statistics of the sample, including means and standard deviations for all the study variables, are presented in **Table 1**.

**Table 1:** Descriptive statistics of the sample.

	n	Mean	SD	Min	Max	α
Age	30	15.9	1.3	12.9	17.9	
BMI	30	16.3	2.5	11.0	20.6	
PSWQ	30	64.43	10.97	43	80	0.896
RRS	30	59.70	14.30	32	88	0.931
MCQ	30	72.27	11.90	47	95	0.823
EDRC	30	58.48	19.81	2	91	0.933

PSWQ, Penn State Worry Questionnaire; RRS, Ruminative Response Scale; MCQ, Metacognitions Questionnaire; EDRC, Eating Disorder Risk composite.

### 3.2 Correlations between worry, rumination, metacognitive beliefs, and eating disorders

Pearson’s *r* coefficients showed that age and BMI were not significantly associated with any of the psychological measures considered (all *p* > 0.05). In contrast, strong and statistically significant positive correlations emerged among the psychological variables. PSWQ scores were highly correlated with RRS. MCQ, and EDRC (all *p* < 0.001). Similarly, RRS showed strong positive associations with MCQ and EDRC (*p* < 0.001 for both). MCQ was also moderately correlated with EDRC (*p* = 0.004). Correlations are presented in **Table 2**.

**Table 2:** Correlations between age, BMI, PSWQ, RRS, MCQ-30, and EDRC.

		Age	BMI (kg/m2)	PSWQ	RRS	MCQ	EDRC
Age	Pearson’s r	—					
	p value	—					
BMI (kg/m2)	Pearson’s r	0.165	—				
	p value	0.385	—				
PSWQ	Pearson’s r	0.185	0.012	—			
	p value	0.329	0.951	—			
RRS	Pearson’s r	0.210	0.056	0.812	—		
	p value	0.266	0.768	<0.001	—		
MCQ	Pearson’s r	0.227	0.206	0.593	0.639	—	
	p value	0.228	0.275	<0.001	<0.001	—	
EDRC	Pearson’s r	0.149	-0.011	0.687	0.742	0.519	—
	p value	0.440	0.954	<0.001	<0.001	0.004	—

BMI, Body Mass Index; PSWQ, Penn State Worry Questionnaire; RRS, Ruminative Response Scale; MCQ, Metacognitions Questionnaire; EDRC, Eating Disorder Risk composite.

### 3.3 Hierarchical linear regression models: worry (PSWQ), rumination (RRS), and metacognitions (MCQ) in predicting eating disorder symptomatology (EDRC)

A hierarchical linear regression model was conducted to examine the role of worry, rumination, and metacognitive beliefs in predicting eating disorder symptomatology.

Age and BMI were not considered potential covariates because they were not significantly associated with the other variables included in the model. Thus, Model 1, including RRS and PSWQ, was significant (F = 17.5, p<0.001) and explained 57% of the variance (R² = 0.574). Within this model, RRS was the only significant predictor (p=0.019), indicating that higher rumination scores were associated with higher EDRC scores. In this model, multicollinearity diagnostics suggested no evidence of severe multicollinearity between the predictors. Both variables exhibited variance inflation factors (2.80) and tolerance values (0.357) within acceptable limits, indicating that multicollinearity is unlikely to represent a major concern.

Model 2 introduced MCQ as a predictor of EDRC. Although the model remained significant overall (F = 11.4, p<0.001), there was no significant incremental variance compared with Model 1 (ΔR² = 0.0034, p=0.658). MCQ was not a significant predictor (p=0.658). The final model explained 58% of variance (R² = 0.578; Cohen’s f^2^ = 1.37). In this model, multicollinearity diagnostics did not indicate severe multicollinearity among the predictors. Variance inflation factors (VIFs) ranged from 1.64 to 3.12, and tolerance values ranged from 0.320 to 0.609. These values fall within commonly accepted limits (VIF < 5; tolerance > 0.10), suggesting that multicollinearity is unlikely to have substantially affected the stability of the regression estimates.

Results are presented in **Table 3**.

**Table 3:** Hierarchical linear regression.

Predictors	B	95%IC		β	SE	p value	R^2^	Adjusted R^2^	ΔR²	F	p value
Model 1							0.574	0.542		17.5	<0.001
RRS	0.749	0.09-0.97	0.535		0.300	0.019					
PSWQ	0.469	-0.18-0.70	0.258		0.389	0.239					
Model 2							0.578	0.527	0.0034	11.4	<0.001
RRS	0.703	0.03-0.97	0.502		0.322	0.038					
PSWQ	0.441	-0.21-0.70	0.242		0.400	0.282					
MCQ	0.130	-0.27-0.41	0.075		0.288	0.658					

RRS, Ruminative Response Scale; PSWQ, Penn State Worry Questionnaire; MCQ, Metacognitions Questionnaire.

Collinearity diagnostics did not indicate severe multicollinearity among the predictors. Variance inflation factors (VIFs) ranged from 1.64 to 3.12, and tolerance values ranged from 0.320 to 0.609. These values fall within commonly accepted limits (VIF < 5; tolerance > 0.10), suggesting that multicollinearity is unlikely to have substantially affected the stability of the regression estimates.

Because the planned sample size was not fully achieved, the regression findings should therefore be interpreted as exploratory and potentially underpowered to detect smaller effects.

In addition, to further address this issue, we conducted a sensitivity analysis to determine the minimum effect size that could be reliably detected with the available sample.

Specifically, assuming a multiple regression model with 3 predictors, an alpha level of 0.05, and a desired statistical power of 0.80, the analysis showed that the study was sufficiently powered only to detect medium-to-large effects (f² = 0.42). This indicates that smaller effects may not have been identified due to the limited sample size, increasing the risk of Type II error.

To further contextualize the findings, we also conducted a *post-hoc* power analysis based on the effect size observed in the final regression model (f² = 1.37). This analysis suggested a high achieved power (0.99), indicating that the observed association was large enough to be detected despite the small sample. However, *post-hoc* power estimates should be interpreted with caution, as they are directly influenced by the observed effect size and do not account for methodological limitations associated with small sample sizes, including reduced generalizability and potential instability of the regression estimates.

## 4 Discussion

The aim of the present exploratory study was to explore the specific association between worry, rumination, and metacognitive beliefs in eating disorder symptomatology in adolescents with AN.

The primary finding showed that rumination significantly accounted for variance in eating disorder symptomatology. This result is consistent with the transdiagnostic model of psychopathology, which conceptualizes worry and rumination as a maladaptive strategy implicated in the maintenance of several disturbances, including EDs (6, 33). Surprisingly, although worry showed strong positive associations with eating disorder symptomatology at the correlational level, it did not maintain an independent predictive role once rumination was included in the regression model. This result suggested that its relationship with symptoms of EDs may be largely overlapping with, or secondary to, ruminative processes (34).

Contrary to our expectations, the addition of metacognitive beliefs to the final regression model did not explain additional variance eating disorder symptomatology, beyond that explained by worry and rumination. The final model showed a very large effect size (Cohen’s f² = 1.37), suggesting strong associations between predictors and eating disorder symptoms in the present sample. However, this estimate should be interpreted cautiously, given the limited sample size and the exploratory nature of the analysis. This finding may reflect the more proximal role of worry and rumination in the association with eating disorder symptomatology, or, alternatively, the possibility that only specific metacognitive domains—rather than overall metacognitive functioning—are relevant in this population. However, the latter hypothesis warrants further investigation.

In the present study, it is also noteworthy that neither age nor BMI was significantly associated with eating disorder symptomatology (at a correlational level) and with any of the psychological variables included in the regression model. This finding is consistent with a growing body of literature suggesting that the severity of ED symptomatology seems to be more strongly associated with cognitive–emotional mechanisms than with demographic or anthropometric indices (35, 36). However, these null findings may reflect limited statistical power, restricted variability, or the use of a global score rather than a specific subscale of the MCQ-30.

Our findings showed that rumination was independently associated with the EDRC score after accounting for worry and metacognitive beliefs, suggesting that rumination may be a relevant cognitive process associated with eating disorder symptomatology in adolescents with AN. From a clinical perspective, these results may support the value of assessing RNT processes in this population. However, given the cross-sectional nature of the study, no causal inferences can be drawn, and it is not possible to determine whether targeting these processes would directly reduce symptoms. Interventions such as Metacognitive Therapy (13, 14), which aim to modify maladaptive metacognitive processes and reduce repetitive thinking, may be relevant in this context, but their applicability to adolescents with AN should be considered preliminary and requires empirical validation.

The present study has several strengths. A major strength of the study is its focus on relatively recent and theoretically relevant constructs. By exploring the associations of worry, rumination, and metacognitive beliefs in relation to eating disorder symptomatology, this study contributes to the growing body of research exploring the underlying cognitive processes of EDs. Moreover, the focus on adolescents addresses a notable gap in the literature, which has largely examined these cognitive processes only in adult samples. An additional strength is the inclusion of participants with AN, which allows for a more specific examination of the role of the investigated processes within this specific eating disorder. However, several limitations of the study must be acknowledged. First, the relatively small and homogeneous sample, composed exclusively of female participants recruited from a single clinical center, limits the generalizability of the findings. Since the planned sample size was not achieved, the regression findings should therefore be interpreted as exploratory. In addition, the small sample size also reduces the ability to detect small-to-moderate effects; thus, some non-significant findings may reflect insufficient sensitivity rather than the absence of true associations.

Second, the cross-sectional design precludes conclusions regarding causal relationships among the study variables. An additional limitation is the absence of measures assessing comorbid anxiety and depressive symptoms. Given the established associations between worry and anxiety, as well as rumination and depression, it would be interesting to assess whether the observed associations with eating disorder symptomatology specifically reflect transdiagnostic repetitive negative thinking processes or are partly attributable to co-occurring internalizing symptoms by including assessments of both anxiety/depressive symptoms and eating disorder-specific repetitive thinking. Furthermore, the exclusive use of self-report measures entails the risk of response bias. In addition, the questionnaires are not specifically tailored for adolescents. However, in a previous study, we used the same version of the PSWQ and RRS with adolescents (25), while other authors have employed the MCQ and EDI-3 in samples of children and adolescents (e.g (37, 38).,.” A further limitation concerns the potential overlap in item content across the self-report measures used in the present study. In particular, some items assessing worry and metacognitive beliefs show partial linguistic and conceptual similarity (e.g., items from the MCQ-30 and the PSWQ referring to difficulties in controlling worry). This overlap may have contributed to inflated associations among measures due to shared method variance rather than reflecting entirely distinct underlying constructs. Therefore, the magnitude of the observed correlations should be interpreted with caution. Finally, using a global metacognition score, rather than specific MCQ subscales, may underdetect the differential contributions of distinct metacognitive domains to eating disorder symptomatology.

Future research employing longitudinal designs and larger, more heterogeneous samples—including male adolescents—is needed to clarify the robustness of these findings and to further delineate the roles of worry, rumination, and metacognitive beliefs in the development and maintenance of eating disorder symptoms during adolescence. Future research should also replicate the study by investigating the specific contributions of each MCQ subscale to clarify the distinct roles of different metacognitive dimensions in eating disorder symptomatology.

## Data Availability

The raw data supporting the conclusions of this article will be made available by the authors, without undue reservation.
